# Evaluation of Antioxidants Using Electrochemical Sensors: A Bibliometric Analysis

**DOI:** 10.3390/s22093238

**Published:** 2022-04-22

**Authors:** Yuhong Zheng, Hassan Karimi-Maleh, Li Fu

**Affiliations:** 1 Institute of Botany, Jiangsu Province and Chinese Academy of Sciences (Nanjing Botanical Garden Memorial Sun Yat-Sen), Nanjing 210014, China; zhengyuhong@cnbg.net; 2 School of Resources and Environment, University of Electronic Science and Technology of China, Xiyuan Ave, Chengdu 610056, China; hassan@uestc.edu.cn; 3 Laboratory of Nanotechnology, Department of Chemical Engineering and Energy, Quchan University of Technology, Quchan 9477177870, Iran; 4 Department of Chemical Sciences, Doornfontein Campus, University of Johannesburg, P.O. Box 17011, Johannesburg 17011, South Africa; 5 Key Laboratory of Novel Materials for Sensor of Zhejiang Province, College of Materials and Environmental Engineering, Hangzhou Dianzi University, Hangzhou 310018, China

**Keywords:** electrochemical sensor, antioxidant, plant extract, polyphenols, flavonoid

## Abstract

The imbalance of oxidation and antioxidant systems in the biological system can lead to oxidative stress, which is closely related to the pathogenesis of many diseases. Substances with antioxidant capacity can effectively resist the harmful damage of oxidative stress. How to measure the antioxidant capacity of antioxidants has essential application value in medicine and food. Techniques such as DPPH radical scavenging have been developed to measure antioxidant capacity. However, these traditional analytical techniques take time and require large instruments. It is a more convenient method to evaluate the antioxidant capacity of antioxidants based on their electrochemical oxidation and reduction behaviors. This review summarizes the evaluation of antioxidants using electrochemical sensors by bibliometrics. The development of this topic was described, and the research priorities at different stages were discussed. The topic was investigated in 1999 and became popular after 2010 and has remained popular ever since. A total of 758 papers were published during this period. In the early stages, electrochemical techniques were used only as quantitative techniques and other analytical techniques. Subsequently, cyclic voltammetry was used to directly study the electrochemical behavior of different antioxidants and evaluate antioxidant capacity. With methodological innovations and assistance from materials science, advanced electrochemical sensors have been fabricated to serve this purpose. In this review, we also cluster the keywords to analyze different investigation directions under the topic. Through co-citation of papers, important papers were analyzed as were how they have influenced the topic. In addition, the author’s country distribution and category distribution were also interpreted in detail. In the end, we also proposed perspectives for the future development of this topic.

## 1. Introduction

In the metabolism process, organisms produce many molecules with high oxidation activity, such as oxygen active free radicals, nitrogen active free radicals, carbon active free radicals, hydrogen peroxide, and singlet oxygen [1]. Excessive concentrations of these molecules can damage biological macromolecules such as DNA and proteins in cells, leading to cancer, cardiovascular disease, and diabetes [2,3,4]. It is well known that plant polyphenols can effectively eliminate free radicals, thus delaying aging and maintaining the health of organisms [5,6,7,8]. These molecules, which can destroy free radicals, are called antioxidants. Therefore, it is of great significance to measure and evaluate antioxidant capacity, or the ability of antioxidants to eliminate active oxidizing substances [9,10]. In addition, antioxidants can prevent or delay food oxidation and can be used as food additives to improve food stability and prolong the storage period [11,12,13,14]. Antioxidants acting on the human body should meet the following conditions: (1) Can react quickly with active oxidation substances; (2) Compared with the active oxidizing substances to be removed, the reaction products are less toxic to cells; (3) Under certain conditions, antioxidants can return to their initial state after removing active oxidation substances and continue to be used to remove active oxidation substances.

Conventional determination methods of antioxidant capacity include chromatography, spectroscopy, and electrochemical methods [15]. Chromatographic equipment is expensive. Although it can distinguish individual antioxidant components in food, it can only give the concentration information and does not realize the accurate evaluation of antioxidant capacity. The spectral method is mainly based on the color of the standard substance and active oxidation substance reaction before and after the change of intensity and the influence of adding an antioxidant to its determination. The spectral method is easily interfered with by the background color because of its determination principle. Therefore, its determination results will have the inevitable error, especially the determination of orange juice, grape juice, and other actual samples. In contrast, electrochemical methods have received much attention in the last decade due to their low cost, simplicity, high sensitivity, and reproducibility [16,17].

In recent years, many research groups have reviewed the evaluation of the anti-oxidation ability of electrochemical sensors. However, these reviews generally cover only electrochemical advances in a particular type of methodology or sensors for specific materials. The achievements and development trends of the whole subject have not been scientifically described. Bibliometrics is a method of statistical analysis of published literature. It can summarize the content of a topic and discuss the trend of various directions under the topic according to its development process, and even predict the future direction. In this work, CiteSpace was used for bibliometric analysis. CiteSpace was developed by Dr. Chaomei Chen, a professor at the Drexel University School of Information Science and Technology [18,19,20,21]. It has become one of the commonly used softwares in bibliometrics analysis. We selected the core collection on Web of Science as a database to assure the integrity and academic quality of the studied material. “Electrochemical antioxidant capacity” has been used as a “topic.” The retrieval period was indefinite, and the date of retrieval was 30 December 2021. After deduplicating the search results, 758 genuine publications were retrieved from 1999 to 2021. CiteSpace 5.8R3 was used to calculate and analyze all documents.

## 2. Developments in the Research Field

### 2.1. Literature Development Trends

**Figure 1:**
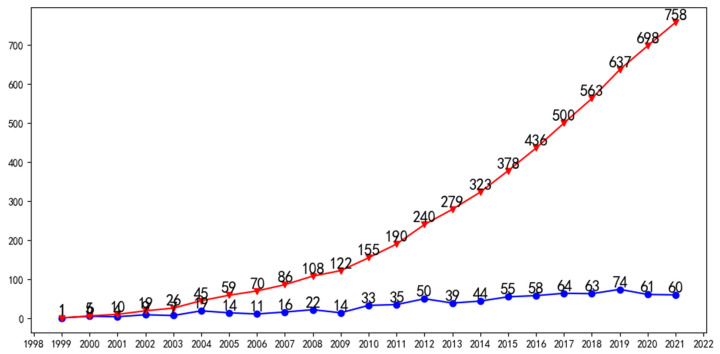
Annual and accumulated publications from 1999 to 2021 about evaluation of antioxidants using electrochemical sensors.

The number of publications on a topic each year is a measure of its popularity. The change in publication numbers can reflect the ability of this topic to attract scholars’ attention in different periods. Figure 1 shows annual and accumulated publications from 1999 to 2021 on the evaluation of antioxidants using electrochemical sensors. The first paper on this topic was published in 1999.

This paper focuses on bone marrow hematopoietic cells [22]. The progenitor cells of bone marrow hematopoietic cells are very vulnerable to acute or chronic oxidative stress. The paper examined high levels of the antioxidant melatonin in rat bone marrow. Since melatonin is an endogenous free radical scavenger and immune enhancer, high melatonin levels in bone marrow cells can provide in situ protection to reduce oxidative damage of these highly vulnerable hematopoietic cells and enhance the immune response capacity of cells such as lymphocytes. In this process, high-performance liquid chromatography-electrochemical detection technology was used. Although electrochemical detection in this technology does not use the same electrochemical sensor as it does today, electrochemical detection in conjunction with chromatography has played a significant role today. The analysis of anti-oxidation abilities by electrochemical technology began before 2000. Chevion et al. [23,24,25,26,27] began using cyclic voltammetry (CV) to evaluate antioxidant capacity in various samples, such as plasma and plant extracts. Elangovan et al. [28] also used CV to evaluate the low molecular weight antioxidant (LMWA) capacity. They determined the total LMWA volumes in plasma and tissues of streptozotocin-induced diabetic rats (1–4 weeks) and insulin-treated diabetic rats.

Between 1999 and 2003, fewer than 10 papers were published on the topic. Starting in 2004, the annual number of papers on the topic exceeded 10 for the first time and stayed above 10 until 2008, when it topped 20. The topic began to attract more attention in 2010, with 33 papers published that year and more than 50 papers 2 years later. Since its inception in 2012, the topic has remained hot. The annual number of publications in 2019 reached a historical peak of 74. The trend in the annual publication numbers shows that the topic has been attracting the attention of scientists since it entered academia. This means that the topic’s concerns continue to influence the academic world, which also means that the topic has not been appropriately addressed. Specifically, no electrochemical sensor can fully meet the evaluation of oxidation resistance.

### 2.2. Journals, Cited Journals, and Research Subjects

**Figure 2:**
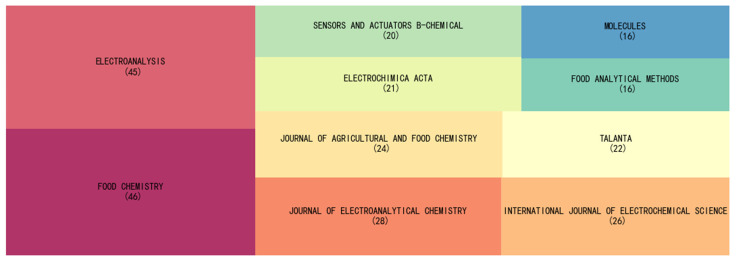
Top 10 journals that published articles on the evaluation of antioxidants using electrochemical sensors.

Figure 2 shows the ten journals with the highest number of publications on evaluating antioxidants using electrochemical sensors. Journals related to food, agriculture, analytical chemistry, electrochemistry, sensors, and molecules are the most frequently published. Food Chemistry and Electroanalysis have published 46 and 45 papers, respectively. Although the detection of antioxidant capacity is a biochemical reaction in vivo, most of the work has not been conducted on in vivo detection. On the other hand, since taking antioxidants is thought to have health benefits, many studies have been reported on food.

Table 1 shows the Top 10 cited Journals. The journals in the table have an excellent agreement with those in Figure 2, such as the Journal of Agricultural and Food Chemistry, Food Chemistry, Talanta, Electroanalysis, etc. This means that these journals not only publish more papers on the topic but also have a broader impact. However, some journals in Table 1 are not in Figure 2, especially Free Radical Biology and Medicine. This is a journal devoted to reports on free radicals. Although the journal does not publish many papers on the detection of antioxidant capacity by electrochemical sensors, its articles are influential and widely cited in other articles. Other classic journals in analytical chemistry, such as Analytical Chemistry and Analytica Chimica Acta, also appear in highly cited journals. Therefore, the establishment and preparation of sensor methodologies are the most concerning direction for this topic.

**Figure 3:**
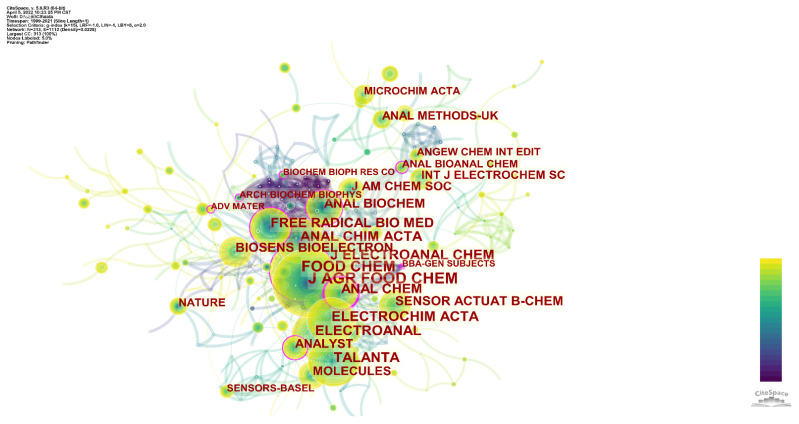
Co-occurrence network of cited journals for evaluation of antioxidants using electrochemical sensors.

Figure 3 shows the Cited Journals network associated with evaluating antioxidants using electrochemical sensors. The whole network can be divided into one large area and one small area. Among them, the most significant area contains the journals listed in Figure 2 and Table 1, representing the most critical journals on this topic. In the upper right corner of the network is a smaller network containing journals in electrochemistry, comprehensive chemistry, and analytical chemistry. The fields of these journals do not differ much from those of the more extensive network. After reading the scope of different journals, we believe that journals in this smaller network pay more attention to methodological innovation of analytical methods. In contrast, the main network focuses more on the actual detection and application potential of antioxidants. Figure 3 shows two other exciting pieces of information. First, there are small nodes with pink circles on the upper part of the more extensive network. The journals represented by these nodes are not very well cited, but they are influential. The links between these journals are purple, indicating that they were significant in earlier years. These journals include Biochemical and Biophysical Research Communications and Archives of Biochemistry and Biophysics.

On the other hand, we can find some very influential journals, such as Nature and Advanced Materials, on the periphery of significant networks. These journals have a very high impact factor and are involved in the topic, but they do not show the same impact on the topic. There are two possible reasons for this. The first is that the articles in these journals have only recently been published and have not been widely cited. The second possibility is that the evaluation of antioxidants using electrochemical sensors appeals only to scholars in a specific field, so reports published in non-chemical journals would not attract much attention.

**Figure 4:**
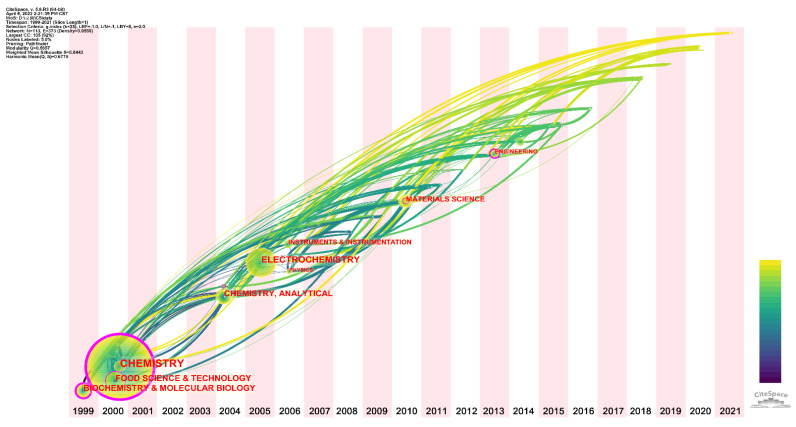
Time-zone view of research categories for the evaluation of antioxidants using electrochemical sensors.

We further analyzed the categories to which all published papers belonged, which can be used to understand where the topic has cross-influence. Figure 4 shows the time-zone view of categories for evaluating antioxidants using electrochemical sensors. Although the focus of this topic is on the evaluation of antioxidation capacity by electrochemical technology, the beginning of this topic covers categories mainly in Chemistry, Food Science & Technology, and Biochemistry & Molecular Biology. In 2004, Analytical Chemistry began to be included in this topic. It was not until 2005 that the first reports were published in a journal belonging to Electrochemistry. This means that electrochemistry was initially used as a standard tool for analysis, and reports did not focus on methodological innovation. This is common in analytical chemistry, where a common technique is first tried to serve a particular purpose. However, this technology often does not fully meet this need and requires methodological innovation. That is why Instruments & Instrumentation has been included in this topic for 2016. Innovation in sensor design and methodology has become an essential part of this topic. In addition, Materials Science was added to the topic in 2010. The discovery and application of new materials can further improve the detection performance of electrochemical sensors. In 2013, Engineering was included in this topic, representing that some of these electrochemical sensors have begun to be experimented with for food industry applications. For the last five years, this topic has been extended to Thermodynamics, Agronomy, Plant Sciences, Green & Sustainable Science & Technology, Polymer Science, and Neurosciences & Neurology.

### 2.3. Geographic Distribution

**Figure 5:**
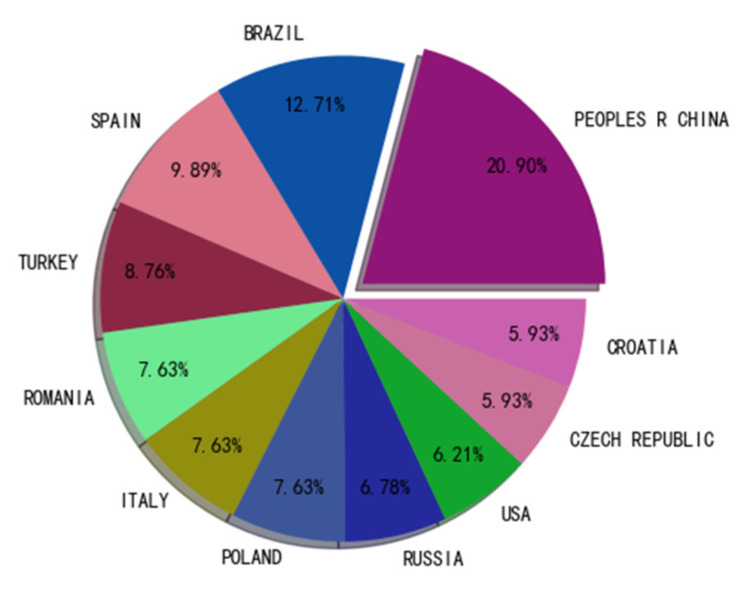
Pie chart of papers published in different countries.

Figure 5 shows the 11 countries with the most publications. As can be seen from the chart, although China contributed 20.90% of publications, it does not mean that it is dominant. In second place was Brazil, contributing 12.71% of publications. Each of the remaining countries contributed less than 10%. As can be seen from the pie chart, this topic has attracted wide attention worldwide, mainly attracting scholars from Asia, Europe, and America. This relatively even distribution of contributions is uncommon in analytical chemistry. Our previous research found that many electrochemically related sensor analyses tend to be concentrated in South Asia, the Middle East, Europe, and South America [29,30].

**Figure 6:**
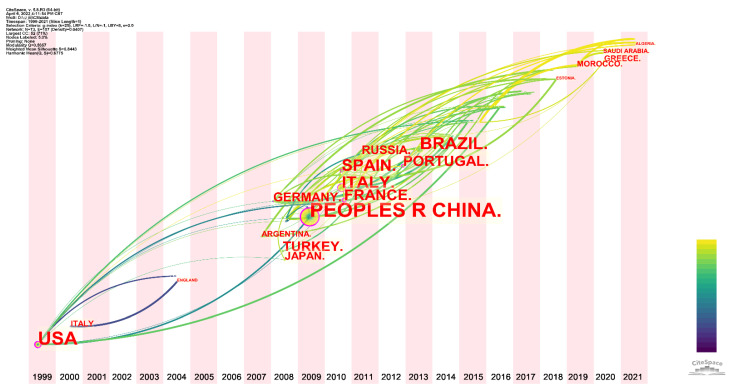
Time-zone view of geographic distribution for the evaluation of antioxidants using electrochemical sensors.

Figure 6 shows the time-zone view of the geographic distribution for evaluating antioxidants using electrochemical sensors. China, Brazil, and Spain contributed the most publications in Figure 5, but the topic was first started by scholars in the USA and Italy in 1999 and 2000, respectively. From the lines connecting different countries, it can be concluded that the work conducted by the US directly influenced subsequent work on the topic. By contrast, the work published in Italy only influenced papers published by British scientists in 2004. Since 2007, several countries have started to pay attention to the topic and become involved. Among them, Argentina became involved in the topic in 2007. Turkey, Germany, and Japan joined the topic in 2008. China became involved in the topic in 2009. Starting in 2010, the topic began to attract more countries. The countries shown in Figure 5 have participated in investigating the evaluation of antioxidants using electrochemical sensors at this stage. The topic continues to attract scientists from several countries, such as Morocco, who started research on the topic in 2019. Saudi Arabia and Greece joined in 2020. Algeria first began investigating the topic in 2021. Combined with the trend of annual publications in Figure 1 and the addition of new countries, this indicates that the evaluation of antioxidants using electrochemical sensors remains very popular and attracts scholars to explore and solve the scientific problems involved.

**Figure 7:**
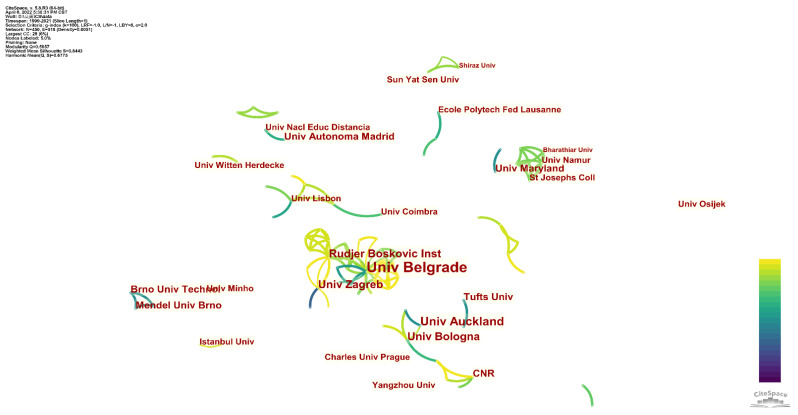
Institution network of published papers for the evaluation of antioxidants using electrochemical sensors.

Given that so many countries are involved in this topic, as shown in Figure 6, a collaboration between different institutions should be frequent. Figure 7 shows the network of cooperation between different institutions. The facts proved inconsistent between this reasonable guess and reality. Collaboration between different institutions to investigate the topic has been limited. The University of Belgrade leads only one extensive collaboration network. There are also two smaller cooperative networks. The first was led by The University of Auckland and The University of Bologna. The other is led by St. Joseph’s College New York and Universite de Namur. The remaining papers do not cover extensive collaboration, neither domestically nor internationally. In particular, the countries that contributed the most papers to the topic did not engage in large-scale cooperation to address it. This is a strange situation, but it can be found in specific research topics. In many cases, the reason is that the content of this topic does not need to be shared by large instruments, nor does it need to collect samples from different regions. As a result, a single research institution can carry out independent investigations.

## 3. Keyword Analysis and Evolution of the Field

Keyword analysis can be used to understand the different research priorities of the topic and reflect on what other topics are closely related to the topic. Table 2 lists the top 20 keywords in this topic. Antioxidant capacity is undoubtedly the keyword with the highest frequency. Keywords related to antioxidant capacity include Capacity, Oxidative stress, Antioxidant activity, and Antioxidant. On the other hand, many keywords are related to electrochemistry and sensors, including Electrode, Behavior, Sensor, Voltammetric determination, Biosensor, and Electrochemical sensor. Some of the remaining keywords were related to specific molecules, including Flavonoid and Polyphenol. They are the two most commonly used to study antioxidant capacity. In addition, Extract is an actual sample often used for antioxidant activity evaluation because some plant extracts are often considered to have excellent antioxidant activity [31,32,33]. Nanoparticle has a high frequency as a keyword because of the development of materials science. Many nanomaterials have been used to construct electrochemical sensors, especially for the surface modification of traditional electrodes [34,35].

Burst detection is a more advanced method than citation counts or downloads for identifying publications receiving significant attention from the research community at various stages of development. Table 3 shows the 11 keywords with the most substantial citation bursts during the research history of the evaluation of antioxidants using electrochemical sensors. Although this topic started in 1999, no burst keyword could be detected before 2004, indicating that this topic did not attract extensive attention from the academic community in the early stage. Disease became the first burst keyword in 2004 and continued until 2013. Antioxidants have long been thought to have health benefits for some chronic diseases [36,37]. How to scientifically measure antioxidant activity has become a challenge in analytical chemistry. Performance liquid chromatography became a burst keyword in 2005. Chromatographic analysis has always played an essential role in medicinal and phytochemical analysis [38]. However, chromatography can only distinguish different molecules to achieve qualitative analysis. Chromatographic and electrochemical detectors enable both qualitative and quantitative analysis [39,40]. For four and five years, Electrochemical detection and Assay became burst keywords in 2009 and 2010. During this period, no other burst keyword appeared, indicating that electrochemical analysis technology began to attract the attention of analytical chemists. A series of works have been devoted to measuring the resistance of electrochemical techniques to oxidation. In 2015, Sample became the new burst keyword, representing that actual samples began to be used to evaluate the previously constructed electrochemical analysis technology and to verify its feasibility. Glassy carbon electrodes became the burst keyword in 2016 and 2017 for a short period. This commercial electrode is widely used in electrochemical sensing because it can be reused after polishing. The emergence of the burst keyword represents that a significant proportion of electrochemical sensors are assembled on glassy carbon electrodes. Since 2018, Nanoparticle and Food have been the burst keywords until now. This indicates that nanomaterials play a significant role in the assembly of electrochemical sensors and have been the focus of scholars’ attention until now. The appearance of Food indicates that the evaluation of antioxidant properties has begun with simple theoretical research to explore potential applications in the food field. The food field is the most direct application scenario of antioxidant capacity evaluation. Different antioxidant capacities of food products can directly affect their price and nutritional value. Vitamin C is one of the most common and synthetic antioxidants found in many foods. Vitamin C is electrochemically active so that electrochemical sensors can detect it. The Vitamin C test became the burst keyword in 2018 and 2019.

**Figure 8:**
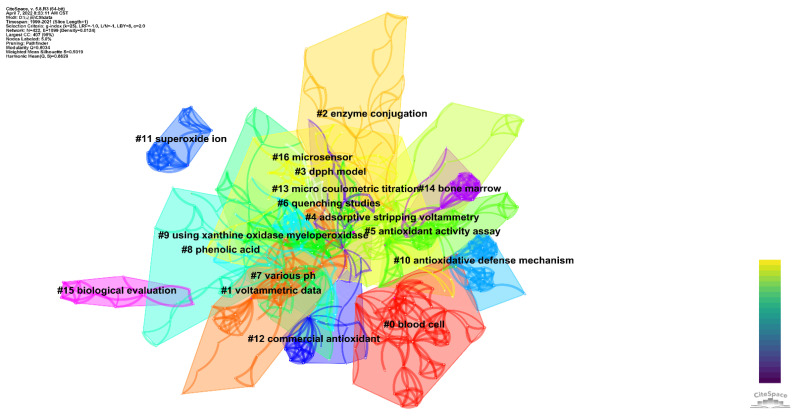
Grouping of keywords for the evaluation of antioxidants using electrochemical sensors.

Cluster analysis of keywords can understand the research focus formed by different keywords in this topic. Figure 8 shows the clustering results of keywords, with 17 clusters formed. Many of these clusters overlap because many publications containing a particular keyword cover other research focuses on this topic. However, some clusters were free from the periphery, which indicated that the focus of these clusters was not directly related to other clusters. Table 4 shows a detailed description of the clusters and their cluster ID, size, and silhouette, as well as the respective keywords. We made a simple interpretation of these clusters based on these keywords and linked publications.

#0 (blood cell) The reports in this cluster mainly describe two types of studies. The first is the harm of oxidative damage to the human body. For example, Silva et al. [41] studied β^s^-haplotypes and Hb F levels of oxidative stress markers in sickle cell anemia in Brazil. Rose et al. [42] examined the levels of biomarkers for oxidative stress in the cerebellum and temporal cortex from autistic patients and unaffected controls. Mice were tested for oxidative damage to the liver caused by morphine [43]. At the same time, ascorbic acid and glutathione can eliminate the damage of morphine to hepatocytes, proving that exogenous antioxidants can protect organs in vivo. The second category is the physicochemical properties of some antioxidants and their effects in vivo. Clavers et al. [44] measured the antioxidant properties of the two chelating agents. CV was used to investigate their structural differences. Sobrova et al. [45] reported the antioxidant capacity of deoxynivalenol. Coenzyme Q10 is a commonly used antioxidant nutraceutical. Since hyperglycemia increases the production of oxygen free radicals, Menke et al. [46] studied the antioxidant levels and redox status of coenzyme Q10 in plasma and blood cells of children with type 1 diabetes. In their investigation, they observed positive results with elevated plasma coenzyme Q10 levels in children with type 1 diabetes compared to healthy children. This contributes to the self-protection of the organism in a state of enhanced oxidative stress. Chen et al. [47] investigated the antioxidant capacity of flavonoids in the almond epidermis (ASF). Their results showed that ASF increased the antioxidant capacity of human low-density lipoproteins to 10 μmol/L Cu^2+^ oxidation-induced ASF. When ASF was combined with vitamin E or ascorbic acid, the effect was better. It is represented that ASF can act synergistically with vitamins C and E to protect low-density lipoprotein from oxidation. This positive result was also verified in hamsters.

#1 (voltametric data) This cluster mainly focuses on the antioxidant properties of plant extracts. Tea [48,49,50] and coffee [51,52,53] are the most frequently studied. That is because any health-related reports from them affect the beverage industry. Extracts from other organisms have also been studied for antioxidant properties, such as brown seaweed *Ascophyllum nodosum* [54], mangrove tannins (*Rhizophora apiculata*) [55], chia (*Salvia hispanica* L.) seeds [56], and lavender [57]. Chromatographic and electrochemical techniques have been compared or combined [48,51,58]. Electrophoresis was also used [59]. However, most of the work uses electrochemical voltammetry technology to detect oxidation.

#2 (enzyme conjugation) This cluster focuses on detecting polyphenols and evaluating antioxidant properties by different detection techniques. Some of this work has focused on specific molecules, such as gallic acid [60,61], chlorogenic acid [62], and catechin [63]. Other analytical methods in this cluster were also used. For example, Marx et al. [64] developed an electronic tongue taste sensor to evaluate the quality of table olives. Mukdasai et al. [65] prepared a new colorimetric paper sensor by modifying filter paper with tetrabutylammonium bromide and sodium dodecyl sulfate. This colorimetric sensor can be used to determine the total antioxidant capacity. The total antioxidant capacity can be determined by how the filter paper changes from yellow to purple. Similarly, Ciou et al. [66] have developed a colorimetric technology-based sensor that quickly detects urinary creatinine.

#3 (DPPH model) This cluster focuses on the mechanisms of antioxidants and the electrochemical detection of antioxidant activity. Brito et al. [67] used UV-VIS spectroscopy to determine the dissociation constant of sesamol. They then investigated the electrochemical behavior of sesamol by controlled potential electrolysis, LSV, and CV. The electrochemical research results were used to interpret its electrochemical redox mechanism and its ability to interact with reactive oxygen species. Marano et al. [68] synthesized two new benzoxazinyl nitro compounds and studied the mechanism of their antioxidant action. CV was used to investigate their electrochemical properties, and their kinetic behavior was studied with the assistance of other techniques. In addition, electrochemical measurements of oxidation capacity were also used to compare with other analytical techniques [69].

#4 (adsorptive stripping volatammetry) Part of the works in this cluster is focused on the combination of electrochemistry and other analytical techniques, including high-performance liquid chromatography [70], flow injection [71], ultraviolet spectrophotometers [72], and electronic tongues [73]. The other part of the works in this cluster focuses on the assembly of electrochemical sensors, especially on the boron-doped diamond electrodes [52,74]. Other reports have focused on detecting polyphenols.

#5 (antioxidative activity assay) The main content of this cluster focuses on investigating hydroxyl radical scavenging by different antioxidants. For example, Ozyurek et al. [75] studied polyphenols and flavonoids’ hydroxyl radical scavenging ability. Bektaşoğlu et al. [76] investigated the hydroxyl radical scavenging capacity of a series of water-soluble antioxidants. Both works use the cupric-reducing antioxidant capacity method for evaluating the hydroxyl radical scavenging effect. The rest reports in this cluster are mainly on investigating the antioxidant capacity of different substances or samples such as rapanone [77], hop *(Humulus lupulus* L.) products [78], and red pigment [79].

#6 (quenching studies) This cluster of reports focused on analyzing the antioxidant properties of flavonoids. In contrast to the previous clusters, several of these reports involve using carbon nanotubes [80,81,82,83]. Carbon nanotubes are a kind of excellent carbon nanomaterial that has aroused great interest in preparing electrochemical sensors. Its excellent electrical conductivity and large comparison area can significantly enhance the sensor’s performance. On the other hand, much of the work in this cluster involves the investigation of adsorption properties [84,85]. At the same time, some work focuses on electron transfer at the interface of electrochemical sensors [86,87].

#7 (various pH) The reports in this cluster mainly investigated the electrochemical behavior of antioxidants, such as catechin [88], bis-coenzyme q(0) [89], gallic acid [90], and Trolox [91]. Different agricultural industries were studied, such as black beans [12], pomegranate juice [81], and grapes [92,93].

#8 (phenolic acid) The main content of this cluster is the synthesis, characterization, and performance analysis of some derivatives of antioxidant molecules. Therefore, the analytical methods of this group are not limited to traditional analytical methods but also use computational chemistry. It is worth noting that the silhouette value in this cluster is only 0.869, so the content concentration within this cluster is relatively low.

#9 (using xanthine myeloperoxidase) Some of the work in this cluster is the preparing of plant extracts and preventing the oxidation of metallic materials [94,95,96,97]. The antioxidants here are not the topic of our work. However, when using keywords to search the literature, the accidental inclusion of some other topics with similar keywords cannot be avoided. On the other hand, some of the work in this cluster has focused on evaluating the antioxidant properties of ascorbic acid (vitamin C) [98,99].

#10 (antioxidative defense mechanism) This cluster contains only two works. Both are case-specific studies. Nia et al. [100] investigated smoking-induced oxidative stress. They used various quantitative oxidative DNA damage and repair markers to determine oxidative stress. At the same time, they also learned about oxidative defense mechanisms. James et al. [101] investigated the intracellular redox status of plasma oxidative stress biomarkers in autistic patients.

#11 (superoxide ion) This cluster contains three reports which analyze the properties of different macromolecules. Feroci and Fini [102] reported interactions between superoxide ions and some sulfur amino acids. Inan et al. [103,104] reported some properties of azo-containing Schiff base ruthenium (II) complexes and azo-azomethine ligands.

#12 (commercial antioxidants) The work of this cluster is also mainly to analyze the properties of some antioxidants. For example, Susana et al. [105] investigated the degradation mechanism of some commercial antioxidants. Rubin et al. [106] investigated the redox mechanism of the coenzyme Q. Poon et al. [107] investigated the relationship between phenoxazine’s free-radical-capturing activities and phenothiazine and temperature.

#13 (micro coulometric titration) This cluster consists of establishing and updating some primary methodologies. For example, Kanyanee et al. [108] reported a simple coulometric titration in a liquid drop. Garcia and Escarpa [109] proposed an electrochemical method based on nickel and nickel-copper nanowires to detect sugar content in honey. Wang et al. [110] proposed a bidirectional indicated redox system to determine o-phenylenediamine.

#14 (bone marrow) There are only three papers in this cluster. The keywords that cluster them together are hydrogen peroxide and in vivo. The paper on H_2_O_2_ was a sensor reported by Emir et al. [111], and the other two works only used this reagent in the experimental process. However, measurements in vivo were conducted by the other two works [22,112].

#15 (biological evaluation) The two works in this cluster investigated the properties of binuclear transition metal complexes [113] and a triphenyltin (iv) 3, 5-dinitrosalicylhydroxamate complex [114].

#16 (microsensor) Only one paper in this cluster reports a quantitative assay for limonin [115]. Cerium dioxide nanoparticles are used to construct an organic electrochemical transistor and detect limonin.

**Table 4:** Knowledge clusters in the field of electrochemical detection of sunset yellow on keyword co-occurrences for each cluster.

Cluster ID	Articles	Silhouette	Keywords	References
0	51	0.977	Antioxidant; Oxidative stress; Disease; Electrochemical detection; Liquid chromatography; DNA damage	[41,42,43,44,45,46,47,116,117,118,119,120,121]
1	40	0.910	Sensor; Antioxidant activity; Performance liquid chromatography; Catechin; Phenolic acid; Capillary electrophoresis	[48,49,50,51,52,53,54,55,56,57,58,59,122,123,124,125,126,127,128,129,130]
2	31	0.909	Nanoparticle; Electrode; Voltammetric determination; Biosensor; Electrochemical sensor; Film	[60,61,62,63,64,65,66,81,131,132,133,134,135,136]
3	29	0.793	Phenolic compound; Cyclic voltammetry; Assay; Ascorbic acid; DPPH; Glassy carbon electrode	[67,68,69,137,138,139,140,141,142,143,144,145,146,147]
4	29	0.915	Polyphenol; Antioxidant capacity; Sample; Wine; HPLC	[52,70,71,72,73,74,148,149,150,151,152,153]
5	26	0.948	Acid; Product; Iron; Neocuproine; Damage; Aromatic hydroxylation	[75,76,77,78,79,154]
6	26	0.947	Antioxidant capacity; Flavonoid; Nanotube; Adsorption; Electron transfer; Protein	[60,70,80,81,82,83,84,85,86,87,97,132,137,138,150,155,156,157,158,159,160,161,162,163,164,165]
7	26	0.976	Oxidation; Behavior; Red wine; Anthocyanin; Expression; Storage	[12,81,88,89,90,91,92,93,166,167,168]
8	25	0.869	Derivative; Caffeic acid; Electrochemical method; Energy; Aqueous solution; Ferulic acid	[169,170,171,172,173,174,175]
9	22	0.943	Capacity; Extract; Vitamin C; Media; Protection; Constituent	[58,67,94,95,96,97,98,99,176,177,178,179,180]
10	20	0.965	Nitric oxide; Lipid peroxidation; Alzheimers disease	[100,101]
11	17	0.992	Antioxidant property; In vitro; Biological activity; Structural characterization; DNA binding	[102,103,104]
12	17	0.983	Mechanism; Graphene oxide; Q(10); Sensitive detection; Inhibition; Carbon electrode	[80,105,106,107,181]
13	16	0.896	Food; Total antioxidant capacity; Tea; Detector	[108,109,110,182,183,184]
14	15	0.949	Hydrogen peroxide; In vivo; Scavenging assay	[22,111,112]
15	11	0.988	Antibacterial activity; By product; Antimicrobial activity; Molecular structure	[113,114]
16	6	0.986	Fruit; Antibacterial	[115]

**Figure 9:**
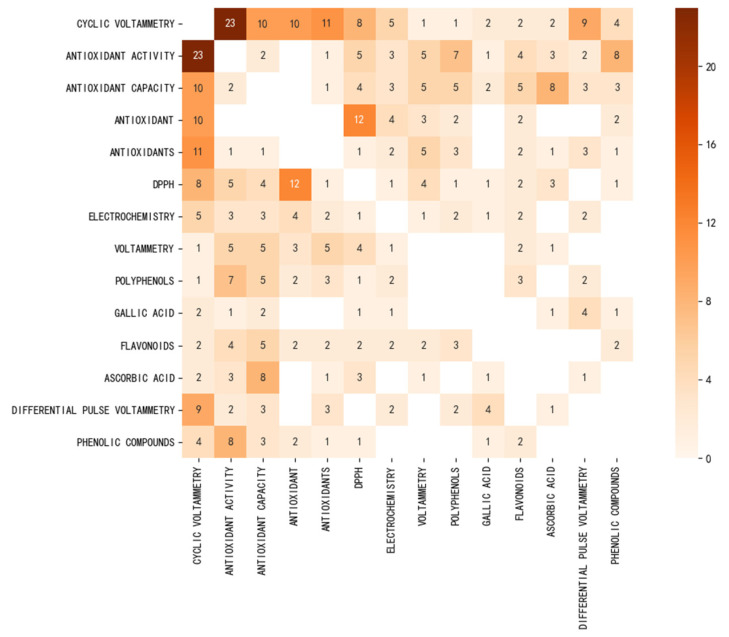
Keywords confusion matrix for the evaluation of antioxidants using electrochemical sensors.

We further use the frequency of occurrence of keywords to make the confusion matrix between keywords (Figure 9). As can be seen from the figure, the most co-occurrence is between cyclic voltammetry and antioxidant activity, indicating that cyclic voltammetry is the most commonly used technique in electrochemistry to measure antioxidant activity. DPPH and cyclic voltammetry also have a strong co-occurrence, indicating that the two techniques can be applied to determine the antioxidant activity of the same substance or sample. DPPH free radical scavenging is a conventional detection technique that should be used as a reference method to measure the accuracy of the proposed electrochemical technology-based sensor. Some antioxidant names are also listed in this co-occurrence diagram, such as polyphenols, gallic acid, flavonoids, and ascorbic acid. The observations here are very consistent with the previous results in keyword analysis. In addition, differential pulse voltammetry also appears. This means that in addition to cyclic voltammetry, differential pulse voltammetry is often used to measure the antioxidant capacity of samples.

Based on the above analysis of keywords, the investigation directions of the evaluation of antioxidants using electrochemical sensors can be summarized as follows:

(1) Cyclic voltammetry and differential pulse voltammetry are the electrochemical techniques most commonly used by electrochemical sensors to analyze the antioxidant capacity of target samples.
(2) The electrochemical behavior of antioxidants can be used to understand the mechanism of these substances during redox.
(3) The primary sources of antioxidants are plants.
(4) Boron doped diamond electrodes, screen printed electrodes, and glassy carbon electrodes are most commonly used as working electrodes for electrochemical sensors.
(5) Carbon nanotubes are the most commonly used nanomaterials for electrode surface modification.

## 4. Co-Citation Analysis

**Figure 10:**
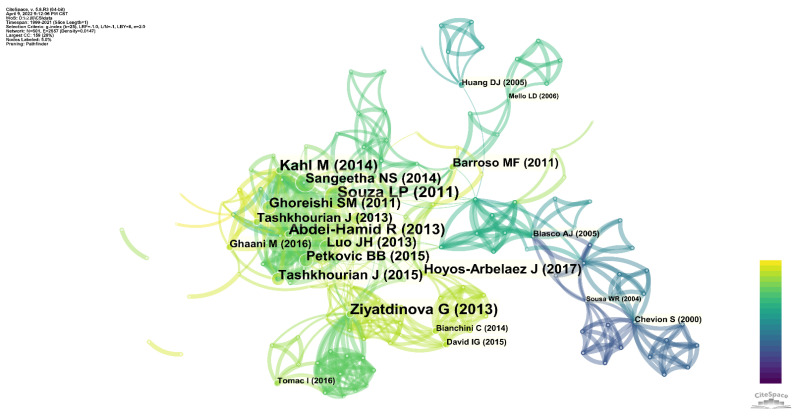
Co-citation analysis for the evaluation of antioxidants using electrochemical sensors.

Co-citation analysis is a critical way to understand the development of the whole topic. Co-citation analysis can learn from the citations of selected data which papers push the topic forward in each direction. Figure 10 shows a co-citation analysis for evaluating antioxidants using electrochemical sensors. As can be seen, the co-citation network is mainly divided into four sub-networks. The largest sub-network on the left side of the figure shows the most influential series of publications in this field.

There are smaller sub-networks at the top and bottom right of the figure, and these networks contain older papers. In the lower right corner of the network, Chevion et al. [27] reported using cyclic voltammetry to measure antioxidant capacity in 2000. Their series of papers is the most pioneering early work on the topic [23,24,25,26,185,186,187]. Sousa et al. [188] detected phenolic antioxidants in orange juice by voltammetry. This work directly affects the largest sub-network in this co-citation network. There is also essential work in this sub-network that connects the research of this period with other work after 2010. Blasco et al. [189] proposed an “Electrochemical Index” to screen samples for “total polyphenolics.” In the upper sub-network, the review by Huang et al. [190] plays an important role. They summarize the chemical theory behind antioxidant capacity assays. This review was used in textbooks on this topic. Similarly, the review by Barroso et al. [191] is also essential. This review summarizes electrochemical sensors for evaluating antioxidant activity, including direct sensing, modified electrode sensing, enzyme sensing, and DNA biosensing. On the other hand, Mello et al. [192] proposed a DNA-electrochemical biosensor to detect antioxidant capacity. The methodology of this sensor is different from conventional electrochemical sensors. DNA damage on the electrode becomes an indicator of antioxidant capacity for such sensors.

Among all sub-networks, the most influential paper reported that the antioxidant capacity of wine was detected by a voltametric scanning technique with carbon nanotube-modified electrodes [193]. Another very influential report describes a novel dual-mediator amperometric sensor for the electrocatalytic oxidation of gallic acid and the reduction of hydrogen peroxide [194]. The papers in this area describe the detection of the antioxidant capacity of some substances or samples by different electrochemical sensors. Ghoreishi et al. [195] used multi-walled carbon-nanotube-modified carbon paste electrodes to detect ellagic acid and gallic acid in *Punica granatum*, *Myrtus communis*, and Itriphal formulations. Tashkhourian et al. [196] reported the detection of gallic acid using a TiO_2_ NPs-modified carbon paste electrode. Similar works were conducted by Luo et al. [197,198] and Petković et al. [199], but the electrode modifier was polyethyleneimine-functionalized graphene, SiO_2_, and a dinuclear copper(II) octaazamacrocyclic complex, respectively. Kahl and Golden [173] deposited a Zn-Al-NO3 layered double hydroxide film on a glassy carbon electrode for the sensing of phenolic acids.

The paper of Ziyatdinova et al. [200] directed this co-citation network to the lower region. This work also fabricated an electrochemical sensor to measure antioxidant capacity, but coffee was used as a study object rather than a specific antioxidant. Tomac et al. [201] detected chlorogenic acids in coffee using differential pulse voltammetry in this sub-network. Bianchini et al. [202] measured caffeic acid in wine. David et al. [203] also proposed a disposable electrode to detect caffeic acid in tea. Therefore, the research content of this smaller network revolves around the content of caffeic acid in coffee and other plants and plant products.

## 5. Conclusions

The evaluation of antioxidant capacity has important application value in medicine and food science. The antioxidant capacity of the product is directly related to its commercial value. Therefore, it is necessary to evaluate antioxidation capacity scientifically and quickly. Traditional analytical techniques can achieve this goal, but they have limitations in practical operation and are not suitable for large-scale promotion. The electrochemical oxidation and reduction properties of antioxidants allow them to be quantitatively and qualitatively evaluated by electrochemical techniques. Based on the above bibliometric analysis, the development and content of this topic are summarized, and the following conclusions can be drawn:

(1) The topic started in 1999 and did not attract much attention until 2010. After 2010, the topic became popular, and the trend continues today. This means that the topic has received much attention so far. At the same time, the problems faced by this theme have not been solved perfectly so far.
(2) In the early stages of this topic, electrochemistry was a quantitative analysis technique, often used in conjunction with chromatographic and spectroscopic techniques for the separation and quantitative detection of complex samples. After that, cyclic voltammetry technology began to attract attention. The electrochemical behavior of antioxidants was used to measure their redox mechanism and quantitatively analyze their antioxidant capacity.
(3) Most antioxidant capacity investigations focus on a specific antioxidant or a group of structurally similar molecules, such as flavonoids and polyphenols. However, due to the availability of plants as an important means for human antioxidant intake, many papers also use electrochemical sensing technology to determine plant samples or plant extracts directly. In addition, antioxidant properties derived from plant extracts can also protect metal materials from corrosion.
(4) Since antioxidants tend to have significant electrochemical redox properties, commonly used commercial electrodes are already capable of direct detection. Carbon paste electrodes, screen printing electrodes, glassy carbon electrodes, and boron-doped diamond electrodes are the most commonly used working electrodes for analysis. However, advances in materials science have greatly improved the performance of electrochemical sensors. Nanomaterial modifications on the surface of the working electrode can improve the sensing performance remarkably. Among them, carbon nanotubes are the materials most used for electrode surface modification in this topic.
(5) Because the oxidants damage DNA, the immobilized DNA on the electrochemical sensor’s surface can be used to measure the antioxidant capacity of antioxidants. The extent to which DNA has been damaged has been an indicator of such electrochemical DNA sensors.

Meanwhile, based on the review of this topic, we believe that the following issues need to be investigated regarding the evaluation of antioxidants using electrochemical sensors:

(1) Direct electrochemical sensors mainly rely on antioxidants’ electrochemical oxidation and reduction behavior. This method helps determine a particular antioxidant, but if the sensor is dealing with a complex sample, the electrochemical behavior is difficult to identify accurately. This is because complex samples contain a series of electrochemically active molecules whose presence can interfere with the measured current value of the target molecule. Therefore, it is a challenge to improve the specificity of direct electrochemical sensors to determine antioxidant capacity.
(2) Choosing suitable electrode modification material is also a significant challenge. The current trend is to modify the electrode by using binary, ternary, or even multiple nanocomposites. Although nanomaterials have excellent properties, the synergistic effect between multiple materials cannot be explained theoretically. Therefore, the performance stability of these nanocomposites has been a limitation to their widespread use. At the same time, the raw materials of some nanocomposites’ preparation are costly and do not have the prospect of mass synthesis.
(3) Although DNA biosensors are methodologically attractive, specially designed DNA requires higher prices. At the same time, such biosensors will be significantly affected by the external environment, so how to ensure their stability is also a meaningful direction.

## Figures and Tables

**Table 1:** Top 10 cited journals with the highest frequency.

No.	Freq	Cited Journal
1	165	Journal of Agricultural and Food Chemistry
2	154	Food Chemistry
3	107	Analytica Chimica Acta
4	102	Talanta
5	102	Journal of Electroanalytical Chemistry
6	94	Electroanalysis
7	91	Electrochimica Acta
8	87	Free Radical Biology and Medicine
9	74	Analytical Chemistry
10	70	Sensors and Actuators B: Chemical

**Table 2:** List of top 20 keywords for the evaluation of antioxidants using electrochemical sensors.

No.	Freq	Centrality	Keywords	No.	Freq	Centrality	Keywords
1	87	0.60	Antioxidant capacity	11	15	0.01	Polyphenol
2	28	0.16	Capacity	12	15	0.02	Electrode
3	23	0.05	Oxidation	13	14	0.11	Behavior
4	22	0.00	Sensor	14	13	0.02	Voltammetric determination
5	20	0.07	Phenolic compound	15	13	0.08	Oxidative stress
6	19	0.29	Antioxidant activity	16	12	0.18	Mechanism
7	17	0.27	Flavonoid	17	12	0.09	Biosensor
8	16	0.21	Acid	18	12	0.05	Electrochemical sensor
9	16	0.15	Antioxidant	19	10	0.03	Derivative
10	15	0.04	Nanoparticle	20	10	0.13	Extract

**Table 3:** 11 keywords with the strongest citation bursts during the research history of the evaluation of antioxidants using electrochemical sensors.

Keywords	Strength	Begin	End	1999–2021
Disease	3.14	2004	2013	▂▂▂▂▂ ▃▃▃▃▃▃▃▃▃▃ ▂▂▂▂▂▂▂▂
Performance liquid chromatography	2.80	2005	2010	▂▂▂▂▂▂ ▃▃▃▃▃▃ ▂▂▂▂▂▂▂▂▂▂▂
Electrochemical detection	3.11	2009	2012	▂▂▂▂▂▂▂▂▂▂ ▃▃▃▃ ▂▂▂▂▂▂▂▂▂
Assay	3.17	2010	2014	▂▂▂▂▂▂▂▂▂▂▂ ▃▃▃▃▃ ▂▂▂▂▂▂▂
Sample	3.55	2015	2018	▂▂▂▂▂▂▂▂▂▂▂▂▂▂▂▂ ▃▃▃▃ ▂▂▂
Glassy carbon electrode	2.60	2016	2017	▂▂▂▂▂▂▂▂▂▂▂▂▂▂▂▂▂ ▃▃ ▂▂▂▂
Nanoparticle	3.28	2018	2021	▂▂▂▂▂▂▂▂▂▂▂▂▂▂▂▂▂▂▂ ▃▃▃▃
Food	2.93	2018	2021	▂▂▂▂▂▂▂▂▂▂▂▂▂▂▂▂▂▂▂ ▃▃▃▃
Vitamin c	2.74	2018	2019	▂▂▂▂▂▂▂▂▂▂▂▂▂▂▂▂▂▂▂ ▃▃ ▂▂
Electrochemical sensor	3.07	2019	2021	▂▂▂▂▂▂▂▂▂▂▂▂▂▂▂▂▂▂▂▂ ▃▃▃
Oxidation	2.87	2019	2021	▂▂▂▂▂▂▂▂▂▂▂▂▂▂▂▂▂▂▂▂ ▃▃▃

## Data Availability

Data sharing not applicable.
